# Development and validation of survival prediction model for gastric adenocarcinoma patients using deep learning: A SEER-based study

**DOI:** 10.3389/fonc.2023.1131859

**Published:** 2023-03-07

**Authors:** Junjie Zeng, Kai Li, Fengyu Cao, Yongbin Zheng

**Affiliations:** Department of Gastrointestinal Surgery, Renmin Hospital of Wuhan University, Wuhan, Hubei, China

**Keywords:** gastric adenocarcinoma, survival prediction, DeepSurv, deep learning, machine learning

## Abstract

**Background:**

The currently available prediction models, such as the Cox model, were too simplistic to correctly predict the outcome of gastric adenocarcinoma patients. This study aimed to develop and validate survival prediction models for gastric adenocarcinoma patients using the deep learning survival neural network.

**Methods:**

A total of 14,177 patients with gastric adenocarcinoma from the Surveillance, Epidemiology, and End Results (SEER) database were included in the study and randomly divided into the training and testing group with a 7:3 ratio. Two algorithms were chosen to build the prediction models, and both algorithms include random survival forest (RSF) and a deep learning based-survival prediction algorithm (DeepSurv). Also, a traditional Cox proportional hazard (CoxPH) model was constructed for comparison. The consistency index (C-index), Brier score, and integrated Brier score (IBS) were used to evaluate the model’s predictive performance. The accuracy of predicting survival at 1, 3, 5, and 10 years was also assessed using receiver operating characteristic curves (ROC), calibration curves, and area under the ROC curve (AUC).

**Results:**

Gastric adenocarcinoma patients were randomized into a training group (n = 9923) and a testing group (n = 4254). DeepSurv showed the best performance among the three models (c-index: 0.772, IBS: 0.1421), which was superior to that of the traditional CoxPH model (c-index: 0.755, IBS: 0.1506) and the RSF with 3-year survival prediction model (c-index: 0.766, IBS: 0.1502). The DeepSurv model produced superior accuracy and calibrated survival estimates predicting 1-, 3- 5- and 10-year survival (AUC: 0.825-0.871).

**Conclusions:**

A deep learning algorithm was developed to predict more accurate prognostic information for gastric cancer patients. The DeepSurv model has advantages over the CoxPH and RSF models and performs well in discriminative performance and calibration.

## Introduction

Gastric cancer remains essential worldwide, with more than 1 million new cases and an estimated 769,000 deaths in 2020 alone, ranking fifth in incidence and fourth in mortality worldwide (1). Notably, the incidence of gastric cancer among young adults worldwide is increasing (2). Adenocarcinoma is the most common subtype of gastric cancer, accounting for 90% of gastric cancer cases (3, 4). The prognosis of gastric cancer varies depending on the type of pathology, molecular subtype, genome, patient’s diet, and physical factors (3). The diversity of prognostic factors provides a challenge for clinicians to predict patient survival based on personal experience accurately.

To improve the precision of lung cancer survival estimations, Cox proportional hazard models and the Kaplan-Meier method have gained popularity in predicting outcomes (5, 6). For example, a nomogram is a reliable tool that can quantify risk by combining and clarifying significant clinical characteristics for clinical oncology. The Kaplan-Meier method uses only the target survival state and time to construct the patient’s survival function (7). However, these traditional models have limitations in the clinical setting of cancer patients, including accurate assessment of overall survival and time to progression. In addition, it is not sufficient to consider only linear relationships between clinical characteristics in clinical decision-making, which does not correspond to the actual clinical situation (8). Therefore, a model that can better account for complex nonlinear variables is needed, which can provide more accurate predictions for clinical decision-making. Accurate prediction of patient survival after diagnosis improves the accuracy of patient prognosis. It might ultimately lead to better-informed decision-making regarding the physician’s and the patient’s family’s efforts to boost a cancer patient’s condition.

Machine learning has more advantages than cox regression models, where the default ending is a simple linear relationship with the variables (9–11). Machine learning is a discipline that focuses on how to make computers learn relationships between data. It allows for constructing unique statistical models from massive data sets that may include hundreds or thousands of data points (12). Machine learning models are built based on machine learning algorithms that can incorporate many variables and data volumes for learning, thus clarifying the complex relationships between variables and outcomes. It is not limited to traditional linear relationships alone. Compared to traditional cox regression models, machine learning predictive models may be more appropriate for the clinical setting and guide clinical decision-making. Artificial neural networks are a subclass of machine learning. Neural networks first process signals in individual neurons and then link different neurons to parameterize the weights of the signals to identify highly complex linear and nonlinear relationships among the input data (13). Deep learning comprises many neural networks that can process more complex information (14).

After reviewing the most relevant advanced studies, we found that many studies have used deep learning models for analytical methods for surgical oncology research. However, most studies have focused on diagnostic applications, such as automated quantification of radiographic images, digital histopathology image interpretation, or biomarker analysis (15–19). To our knowledge, there are few examples of published studies using deep learning models for prognostic prediction in surgical oncology. In gastric cancer research, deep learning techniques have been applied to digital histopathology image interpretation and image feature discrimination. However, to our knowledge, only a few studies have focused on predicting the survival of gastric cancer patients. As an algorithmic structure, neural networks can receive a large amount of feature information and learn the correlation between features, including complex nonlinear relationships. Deep learning networks are the superposition of multiple neural network structures, and this model explains the complex linear and nonlinear relationships between variables. Katzman et al. developed a novel deep learning method using a deep learning network to integrate Cox proportional hazards for survival analysis, referred to as the deep learning survival neural network (DeepSurv) (20). The authors show that the deepsurv model can achieve the same, if not superior, performance as the traditional published survival model.

This study aimed to develop models for predicting the survival of patients with gastric adenocarcinoma using the deep learning survival neural network and compared the predictive performance with other standard survival models. Expect a best-in-class model to provide accurate survival predictions for clinical decision-making.

## Method

### Data source

**Figure 1:**
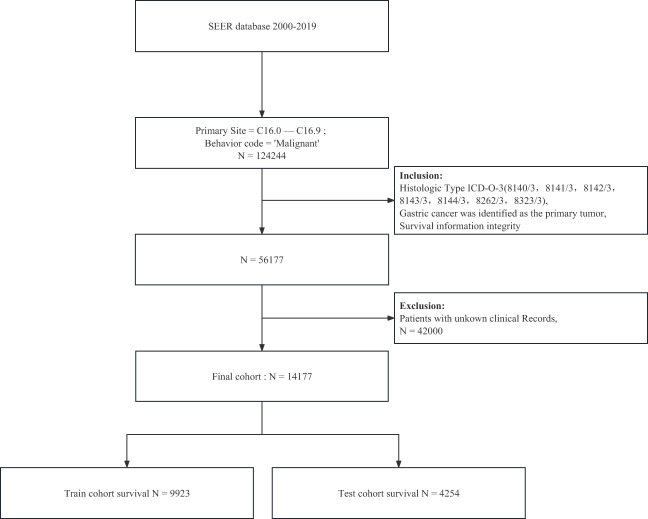
The flow diagram of patients with gastric adenocarcinoma selection.

The Surveillance, Epidemiology, and End Results (SEER) database is publicly available nationwide. Searched the database for gastric cancer cases and their corresponding details between 2000-2019 using SEER*Stat version 8.4.0 software, which contains 17 data centres. First, patients with cancer at the primary site of the stomach were retrieved based on the location code and tumor nature code in the International Classification of Diseases of Oncology. Furthermore, the tumor was ensured to be the first primary tumor of the patient based on the frequency codes provided in the SEER database. Second, to focus on patients with adenocarcinoma, we included only patients aged >20 with ICD-O-3 tissue/behavior codes 8140/3, 8141/3, 8142/3, 8143/3, 8144/3, 8262/3, and 8323/3, ensured that they had complete follow-up information, for a total of 56,177 patient information. Then we removed the cases with reliable information according to the variables we included. A flowchart displaying the detailed selection process is presented in **Figure 1**.

### Variable’s definitions

The following parameters were collected from the sample: age at diagnosis, sex, race, marital status, site of the primary tumor, pathological grade, Summary Stage, pathological primary tumor T stage according to AJCC 7th edition (T0-T2/T3/T4/unknown-NA), pathological according to AJCC 7th edition primary tumor lymph node staging (N0/N1/unknown-NA), pathological primary tumor metastasis information according to AJCC 7th edition, AJCC staging, targeted surgical resection of all visually visible cancer sites (yes/no), regional lymph node dissection information, chemotherapy information, radiotherapy information, Months from diagnosis to treatment, number of lymph node biopsies, number of positive lymph node biopsies, tumor size (based on the largest tumor diameter), presence of bone metastases, brain metastases, lung metastases, liver metastases, overall survival time and disease-specific deaths. After screening, we only used the information of patients diagnosed from 2004-2015 because the information outside this period had some missing data. These missing data include complete information on radiotherapy, chemotherapy, and tumor size, which are essential for our model building.

### Model development

**Figure 2:**
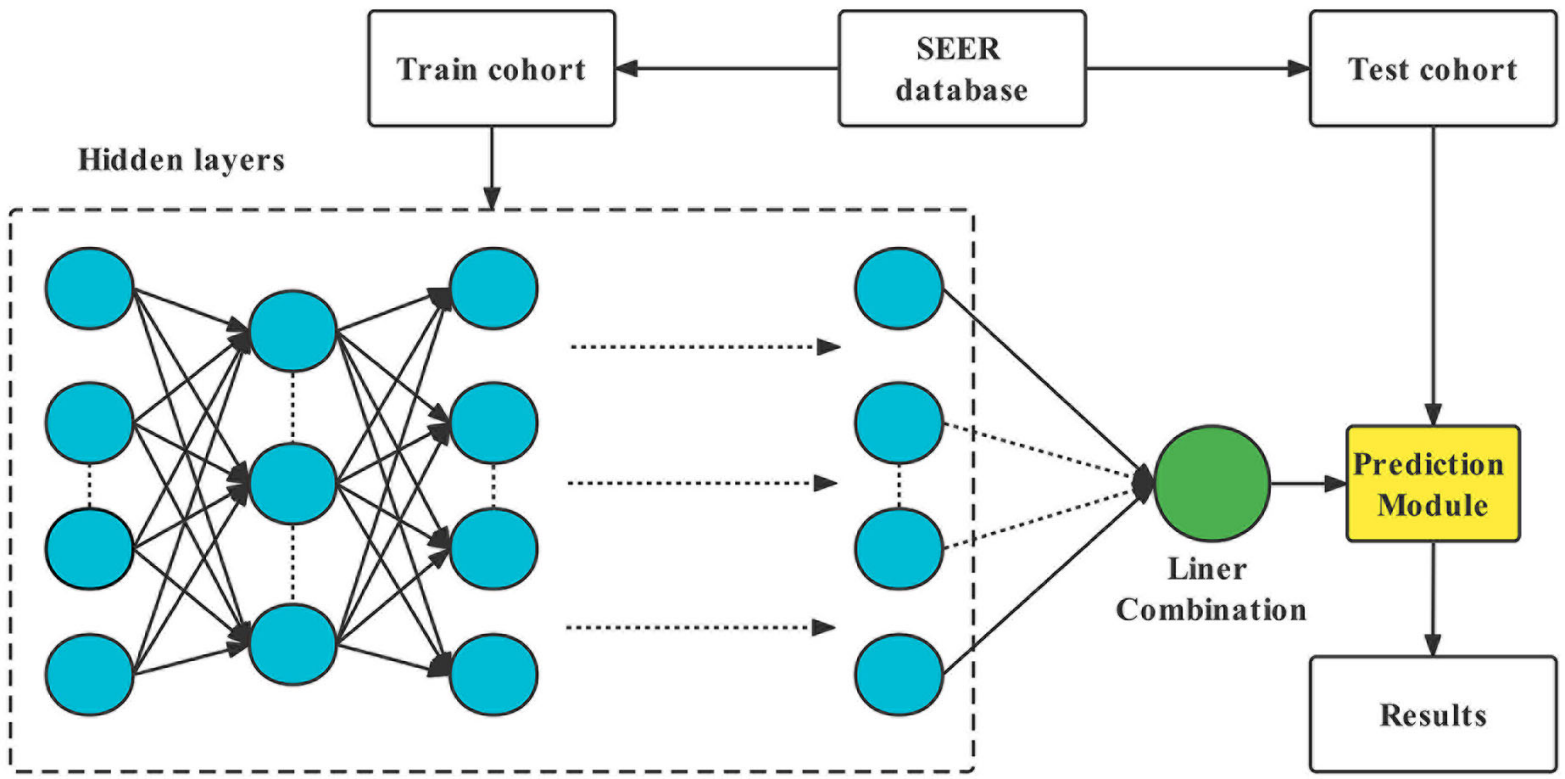
Diagram of the deep learning procedure.

The random grouping of datasets relied on the sklearn package in python. The function “sklearn.model_selection” was applied to randomly divide all patients into training and test cohorts with a ratio of 7:3. Two algorithms - one based on neural networks (DeepSurv) and one based on machine learning (RSF) - were selected for training. A multivariate CoxPH model was also constructed for comparison. DeepSurv is a deep feed-forward neural network that can be applied to survival prediction. The network consists of many neurons, divided into three main parts: an input layer, an output layer, and a hidden layer (10, 20). The graphic representation of DeepSurv is given in **Figure 2**. Additional information on model training is shown in the **Supplementary Material**.

### Model evaluation

The C-index, a correlation coefficient between anticipated survival risks and actual survival times, was used to assess the models’ accuracy. A C-index value of 0.5 denotes a random prediction. In contrast, a C-index value of 1.0 denotes an accurate forecast. Kang’s approach was used to determine whether the C-index of the two models differed. Additionally, Brier scores—which range from 0 to 1, with 0 being the best outcome—were obtained. They represent the mean square difference between the observed patient state and the expected survival probability. In practice, a model is deemed helpful if its Brier score is less than 0.25. To measure the overall validity of the model over all available periods, an Integrated Brier Score (IBS) was also generated. The 1-, 3-, 5-, and 10-year O.S. were calibrated using a calibration curve to compare anticipated and actual survival. Receiver operating characteristic (ROC) curves were produced, and area under the curve (AUC) values were computed for 1-, 3-, 5-, and 10-year survival to evaluate the time-dependent sensitivities and specificities of the models. The prediction model is then trained using the training data, and after several iterations, the algorithm determines the best learning rate and least amount of value loss.

### Statistical analysis

A basic statistical description of the data was performed using the R programming language (https://www.r-project.org/). U-tests for continuous variables and chi-square tests for categorical variables were used to assess baseline differences between the training and test sets. This study used Python software (https://www.python.org/) to perform the other calculations and analyses. Cox regression models were built based on the lifeline package for python. For the K-M survival analysis in this study, the machine learning and survival learning models are built based on python’s sick-survival 0.19.0 package (21). Python’s PyTorch package does the construction of deep learning models (22). The data visualization is done by GraphPad Prism 9 (https://www.graphpad-prism.cn/) and python.

## Results

### Basic characteristic

A total of 14177 individuals with gastric adenocarcinoma reported in the SEER database between 2004 and 2015 were included in the research. The primary patient characteristics are shown in **Table 1**. 9742 cases were female (69%), and 4435 were male (31%); 11660 cases were 20-80 years old (82%), and 2517 cases were 80+ years old (69%). The predominant race of the case species included in the study was white (69%), and 9083 cases were married (64%). The majority of tumors were in C16.0 (37%), grade III/IV (58%), and AJCC stage I (28%). 11121 cases underwent resection of the primary tumor (78%), and 3080 cases did not receive surgical treatment (22%). The dataset was randomly divided into the training cohort (n = 9923) and testing cohort (4254) at a ratio of 7:3. For each variable, there were no significant changes between the training cohort and the test cohort. There were also no survival differences between the two groups (*p* = 0.28).

**Table 1:** Clinical and pathological features of patients with gastric adenocarcinoma.

Characteristics	Train cohort	Test cohort	p-value
	(n=9923)	(n=4254)	
Age			0.36
20-80 years	8181(82.44%)	3479(81.78%)	
80+ years	1742(17.56%)	775(18.22%)	
Sex			0.85
Female	6824(68.77%)	2918(68.59%)	
Male	3099(31.23%)	1336(31.41%)	
Race			0.23
American Indian/Alaska Native	65(0.66%)	41(0.96%)	
Asian or Pacific Islander	1837(18.51%)	770(18.10%)	
Black	1183(11.92%)	496(11.66%)	
White	6838(68.91%)	2947(69.28%)	
Marital status			0.83
Married	6353(64.02%)	2715(63.82%)	
Unmarried	3570(35.98%)	1539(36.18%)	
Primary Site^*^			0.46
C16.0	3703(37.32%)	1591(37.40%)	
C16.1	341(3.44%)	127(2.99%)	
C16.2	767(7.73%)	350(8.23%)	
C16.3	2179(21.96)	897(21.09%)	
C16.4	356(3.59%)	141(3.31%)	
C16.5	961(9.68%)	400(9.40%)	
C16.6	400(4.03%)	187(4.40%)	
C16.8	607(6.12%)	281(6.61%)	
C16.9	609(6.14%)	280(6.58%)	
Grade			0.90
Grade I/II	4200(42.33%)	1806(42.45%)	
Grade III/IV	5723(57.67%)	2448(57.55%)	
Summary Stage			0.62
Distant	2368(23.86%)	1031(24.24%)	
Localized	2768(27.89%)	1209(28.42%)	
Regional	4787(48.24%)	2014(47.34%)	
T stage			0.25
T1	2299(23.17%)	995(23.39%)	
T2	2798(28.20%)	1252(29.43%)	
T3	2930(29.53)	1178(27.69%)	
T4	1446(14.57%)	635(14.93%)	
TX	450(4.53%)	194(4.56%)	
N stage			0.53
N1	3917(39.47%)	1712(40.24%)	
N2	3522(35.49%)	1520(35.73%)	
N3	1397(14.08%)	552(12.98%)	
N4	863(8.70%)	374(8.79%)	
N.X.	224(2.26%)	96(2.26%)	
M stage			0.74
M0	7757(78.17%)	3312(77.86%)	
M1	2150(21.67%)	937(22.03%)	
MX	16(0.16%)	5(0.12%)	
AJCC stage			0.68
I	2787(28.09%)	1205(28.33%)	
II	2121(21.37%)	924(21.72%)	
III	2499(25.18%)	1030(24.21%)	
IV	2516(25.36%)	1095(25.74%)	
Surgery of the primary site			0.42
No	2131(21.48%)	940(22.10%)	
Yes	7792(78.52%)	3314(77.90%)	
Lymph node dissection			0.41
No	2652(26.73%)	1170(27.50%)	
Yes	7271(73.27%)	3089(72.61%)	
Radiation recodes			0.67
No	6341(63.90%)	2735(64.29%)	
Yes	3582(36.10%)	1519(35.71%)	
Chemotherapy			0.51
No	4370(44.01%)	1847(43.42%)	
Yes	5553(55.96%)	2407(56.58%)	
Months from diagnosis to treatment			0.52
Mean (S.D.)	1.03(1.14)	1.05 (1.19)	
Median [Min, Max]	1.00 [0, 23.0]	1.00 [0, 20.0]	
Regional nodes examined			0.13
Mean (SD)	14.4 (17.2)	14.5 (17.7)	
Median [Min, Max]	11.0 [0, 99.0]	11.0 [0, 99.0]	
Regional nodes positive			0.38
Mean (SD)	28.3 (41.6)	27.8 (41.4)	
Median [Min, Max]	4.00 [0, 99.0]	3.00 [0, 99.0]	
Tumor bone metastasis			0.28
No	9815(98.91%)	4198(98.68%)	
Yes	108(1.09%)	56(1.32%)	
Tumor brain metastasis			1.00
No	9898(99.75%)	4244(99.76%)	
Yes	25(0.25%)	10(0.24%)	
Tumor liver metastasis			0.10
No	9380(94.53%)	3991(93.82%)	
Yes	543(5.47%)	263(6.18%)	
Tumor lung metastasis			0.32
No	9766(98.42%)	4176(98.17%)	
Yes	157(1.58%)	78(1.83%)	
Tumor size			0.57
< 1cm	9645(97.20%)	4151(97.58%)	
< 2cm	7(0.07%)	2(0.05%)	
< 3cm	16(0.16%)	3(0.07%)	
< 4cm	16(0.16%)	7(0.16%)	
< 5cm	8(0.08%)	5(0.12%)	
≥ 5cm	231(2.33%)	86(2.02%)	

* Primary Site, this data item identifies the site in which the primary tumor originated. See the International Classification of Diseases for Oncology, Third Edition (ICD-O-3) for topography codes. The decimal point is eliminated. C16.0, Cardia; C16.1, Fundus of stomach; C16.2, Body of stomach; C16.3, Gastric antrum; C16.4, Pylorus; C16.5, Lesser curvature of stomach NOS; C16.6, Greater curvature of stomach NOS; C16.8, Overlapping lesion of stomach; C16.9, Stomach.

### Model comparisons

**Figure 3:**
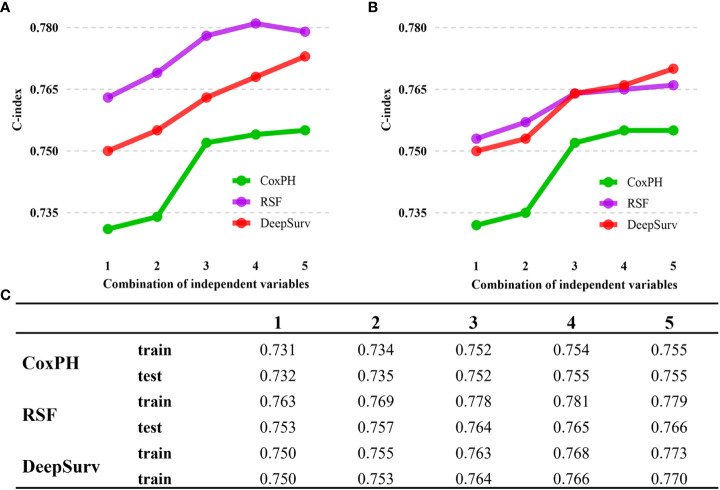
C-index performance of the DeepSurv, RSF, and COXPH models. **(A)** C-index performance on train cohort. **(B)** C-index performance on train cohort. **(C)** Summary of C-index for each models. DeepSurv fared the best of the three models, displaying a considerably more positive trend. The numbers 1 to 5 indicate the different variable compositions. The addition of statistically insignificant factors is seen in Points 4 and 5. Point 1 indicates the Age, Race, Marital status, Summary Stage, T stage, M stage, Surgery of the primary site, regional nodes examined, regional nodes positive, Tumor bone metastasis, and Tumor size. Point 2 adds new variables, including Primary Site and Grade. Point 3 adds variable N stage, AJCC stage, and chemotherapy. Point 4 continues to add variables Months from diagnosis to treatment and sex. Point 5 then adds the remaining insignificant variables from the cox analysis.

**Figure 4:**
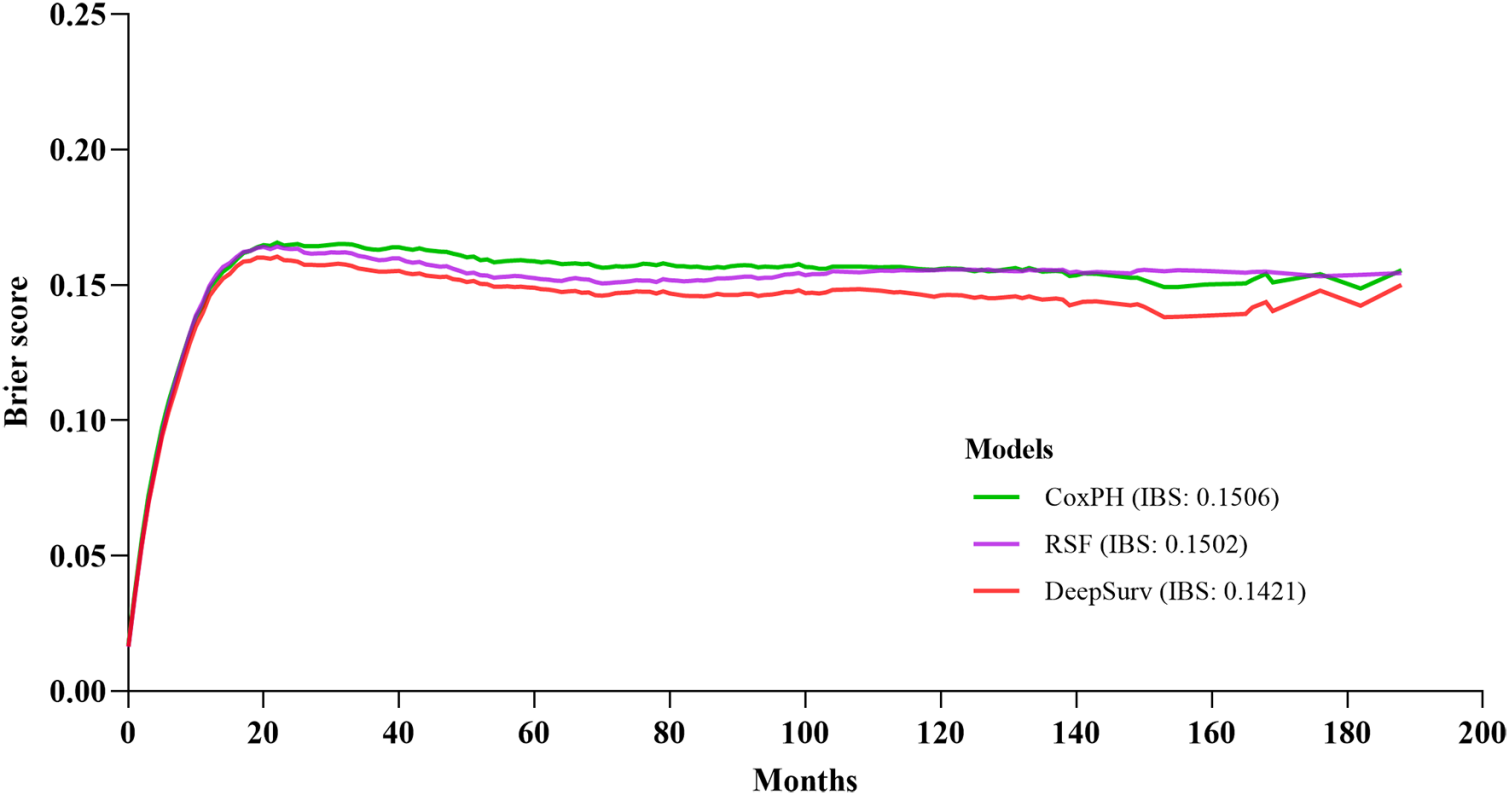
Prediction error curve. A useful model will have a Brier score less than 0.25 as a standard.

With the training data, survival models were created based on CoxPH regression, Random Survival Forest (RSF), and DeepSurv (a deep learning-based model). The performance of these three models was evaluated by comparing Harrell’s c-index, which assesses the agreement between anticipated hazards and actual survival, applied to both the training and testing set. The three models performed differently, with DeepSurv’s c-index on testing sets reaching 0.770, RSF 0.766, and the CoxPH model 0.755. The characteristics gradually increased from eleven to twenty-three. In CoxPH regression, the first eleven characteristics were statistically significant variables (**Tables 2**, **3**). Subsequently, more statistically significant and unimportant characteristics were added. While RSF and CoxPH models did not exhibit the steady increasing trend when statistically inconsequential characteristics (sex, radiation recodes, tumor liver metastasis, brain metastasis, lung metastasis, and Lymph node dissection) were introduced, DeepSurv’s c-index did as the features were added one at a time (**Figure 3**). Although these factors were statistically unimportant in the CoxPH study, they are nevertheless thought to be crucial for prediction and decision-making in a clinical environment. The IBS of the three models were 0.142 (DeepSurv), 0.150 (RSF), and 0.151 (CoxPH) (**Figure 4**).

**Table 2:** Univariate CPH analysis.

Covariate	H.R.	95%CI	p
Age	1.24	1.16-1.33	< 0.05
Sex	0.92	0.87-0.97	< 0.05
Race	1.14	1.11-1.18	< 0.05
Marital status	1.13	1.07-1.19	< 0.05
Primary Site	0.99	0.98-1.00	0.03
Grade	1.68	1.59-1.77	< 0.05
Summary Stage	0.68	0.66-0.70	< 0.05
T stage	1.62	1.58-1.65	< 0.05
N stage	1.52	1.49-1.56	< 0.05
M stage	3.84	3.63-4.05	< 0.05
AJCC stage	2.02	1.97-2.07	< 0.05
Surgery of the primary site	0.29	0.27-0.30	< 0.05
Chemotherapy	1.49	1.41-1.57	< 0.05
Months from diagnosis to treatment	0.88	0.86-0.91	< 0.05
Regional nodes examined	0.99	0.99-0.99	< 0.05
Regional nodes positive	1.01	1.01-1.01	< 0.05
Tumor bone metastasis	4.30	3.53-5.24	< 0.05
Tumor size	1.20	1.17-1.24	< 0.05
Lymph node dissection	0.39	0.37-0.42	< 0.05
Tumor liver metastasis	3.29	3.00-3.62	< 0.05
Radiation recodes	1.06	1.01-1.12	0.02
Tumor brain metastasis	4.31	2.91-6.39	< 0.05
Tumor lung metastasis	3.67	3.11-4.34	< 0.05

**Table 3:** Multivariate CPH analysis.

Covariate	H.R.	95%CI	p
Age	1.37007	1.28-1.47	< 0.05
Sex	0.943397	0.89-1.00	0.06
Race	1.087829	1.05-1.13	< 0.05
Marital status	1.13522	1.07-1.20	< 0.05
Primary Site	0.981495	0.97-0.99	< 0.05
Grade	1.319365	1.25-1.39	< 0.05
Summary Stage	1.148254	1.08-1.22	< 0.05
T stage	1.080254	1.05-1.1	< 0.05
N stage	1.13535	1.10-1.17	< 0.05
M stage	1.310824	1.13-1.52	< 0.05
AJCC stage	1.629648	1.55-1.71	< 0.05
Surgery of the primary site	0.595993	0.52-0.69	< 0.05
Chemotherapy	0.625112	0.58-0.67	< 0.05
Months from diagnosis to treatment	0.904289	0.88-0.93	< 0.05
Regional nodes examined	0.996188	0.99-1.00	< 0.05
Regional nodes positive	1.004607	1.00-1.01	< 0.05
Tumor bone metastasis	1.286578	1.05-1.58	< 0.05
Tumor size	1.043229	1.01-1.07	< 0.05
Lymph node dissection	1.111767	0.92-1.34	0.26
Tumor liver metastasis	0.946553	0.85-1.05	0.31
Radiation recodes	0.999806	0.94-1.06	1.00
Tumor brain metastasis	1.375527	0.92-2.05	0.12
Tumor lung metastasis	1.13761	0.95-1.36	0.15

**Figure 5:**
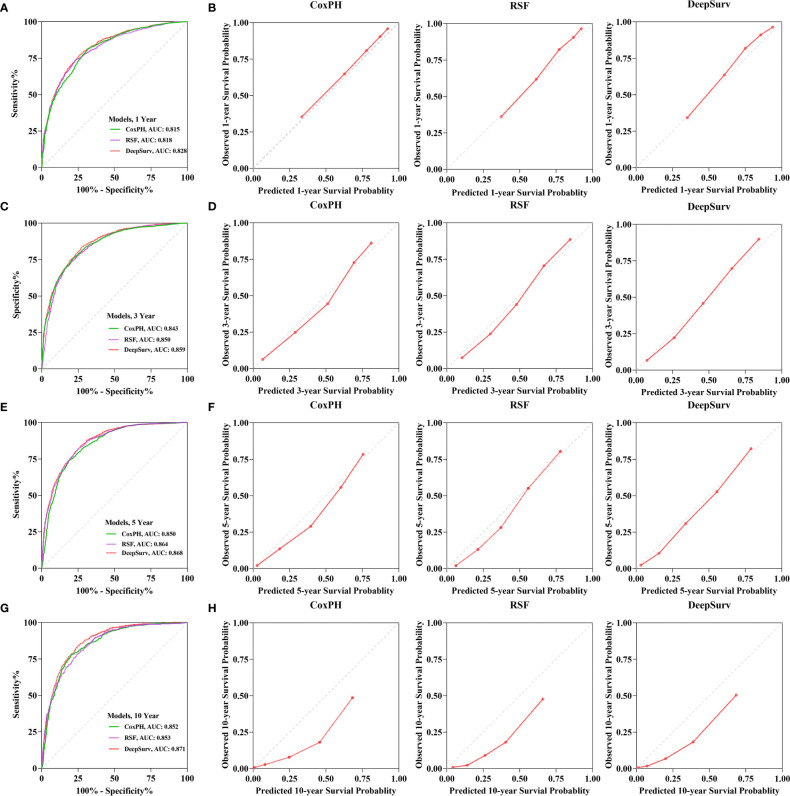
The receiver operating curves (ROC) and calibration curves for 1-, 3-, 5-, 10-year survival predictions. ROC curves for **(A)** 1-, **(C)** 3-, **(E)** 5-, **(G)** 10-year survival predictions. calibration curves for **(B)** 1-, **(D)** 3-, **(F)** 5-, **(H)** 10- year survival predictions.

The calibration plots demonstrated that the DeepSurv model, followed by the CoxPH, RSF, and 1-, 3-, 5-, and 10-year overall survival rates, had the highest concordance between model prediction and actual observation (**Figure 5**). The AUC was more prominent for the DeepSurv model than for the three other models (1-year-AUC of DeepSurv: 0.828, RSF:0.818, CoxPH: 0.815; 3-year-AUC of DeepSurv: 0.859, RSF: 0.850, CoxPH: 0.859; 5-year-AUC of DeepSurv: 0.868, RSF: 0.864, CoxPH: 0.850; 10-year-AUC of DeepSurv: 0.871, RSF: 853, CoxPH: 0.852) (**Figure 5**). The results demonstrated that compared to RSF and traditional CoxPH models, deep learning models, particularly the DeepSurv model, were more reliable in predicting the survival prognosis of patients with gastric adenocarcinoma.

### Feature importance

**Figure 6:**
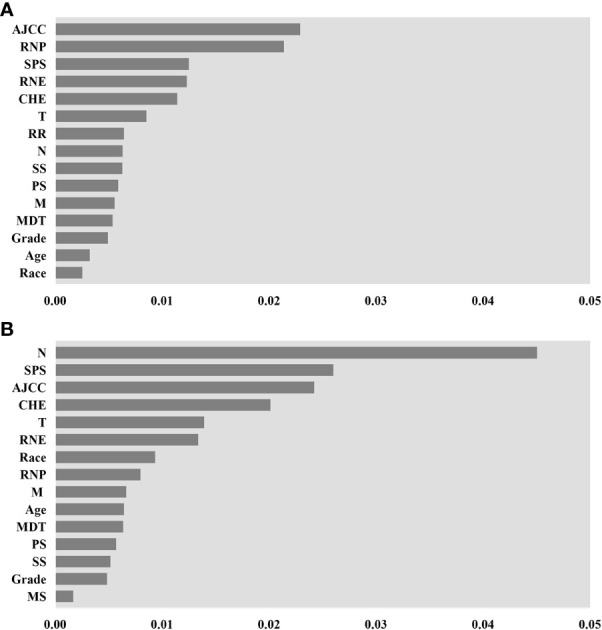
Feature importance for DeepSurv and random survival forest (RSF) models, only the top 15 variables in importance are shown in the figure. **(A)** is the importance of features measured by the RSF model. **(B)** is by DeepSurv. AJCC stage (AJCC), Regional nodes positive (RNP), Surgery of the primary site (SPS), Regional nodes examined (RNE), Chemotherapy (CHE), T stage(T), radiation recodes (R.R.), N stage (N), Summary Stage (S.S.), Primary Site (P.S.), M stage (M), Months from diagnosis to treatment (MDT), Marital status (M.S.).

The assessment of feature importance identified features important to model accuracy for prognosis. For the DeepSurv model and RSF model construction, the features ranked in the top 15 in importance are shown in **Figure 6**. For the RSF model, AJCC staging, positive regional nodes, primary site surgery, regional node examination, and chemotherapy are located at the top. The importance ranking measured by the DeepSurv model differs from that of the RSF model.

## Discussion

For patient counselling, follow-up, and therapy planning, accurate prediction of gastric adenocarcinoma survival is essential. Previous research has shown that several prognostic markers, such as patient age, tumor size, histological type, tumor grade, and metastasis, can affect a patient’s chance of surviving after being diagnosed with gastric adenocarcinoma. In parallel, genetic and imaging data is being analyzed for gastric adenocarcinoma patient survival. The limits of the linear relationship between variables anticipated by the traditional CoxPH model become clear in high-dimensional data. Because deep learning can completely disclose potential nonlinear relationships in data, it is used in survival analysis. This technique has been successfully used to analyze clinical, imaging, and genetic data in recent years. As far as we know, this approach has not been applied to gastric adenocarcinoma. In order to predict the survival of patients with gastric adenocarcinoma, we created one deep-learning model and evaluated its performance against two conventional models.

This study developed various models for predicting the survival of patients with gastric adenocarcinoma using data from the SEER database. The neural network DeepSurv model performed the best, followed by RSF and CoxPH.The training dataset’s C-index value for the DeepSurv model was 0.773, while the test dataset’s value was 0.770. There is a slight difference between the values of the three models on the C-index. We reviewed the relevant literature, and the gap between their models` c indices was between 0.005 and 0.024 (23–26). Therefore, the DeepSurv model is advantageous in predicting the survival rate of gastric adenocarcinoma patients. DeepSurv’s performance in discrimination and calibration for projecting 1-, 3-, 5-, and 10-year survival was further evaluated by ROC and calibration curves. When dealing with huge samples, many variables, and nonlinearity, the DeepSurv model outperforms previous models by using deep learning techniques to represent the probability of occurrences as a function of time.

In this study by gathering afflicted individuals who resided in the United States from the SEER database, this study created a DeepSurv model of the survival rate of patients with stomach adenocarcinoma. In order to determine risk variables for the prognosis of 9923 patients with gastric adenocarcinoma in the training cohort, we first performed a Cox proportional-hazards regression analysis. Age, race, marital status, tumor grade, primary site, AJCC TNM stage, summary stage, chemotherapy, tumor size, months from diagnosis to treatment, primary site surgery, regional nodes examined, positive regional nodes, information on tumor bone metastasis, radiation recodes, and grade were among these risk factors (*p*<0.05) (**Table 3**). The remaining six variables (included Sex, Radiation recodes, Tumor brain metastasis, Tumor lung metastasis, lymph node dissection) although exhibited as non-significant variables in the CoxPH regression analysis (p>0.05) do assist in the predictive performance of the DeepSurv model (**Figure 3**). This may be due to the superiority of deep learning algorithms. Input, hidden, and output layers comprise the three-layer network structure used by the DeepSurv technique (27). The hidden layer has a multilayer structure for variable conversion, and the output layer is the converted target variable. The input layer contains each linear or nonlinear predictor variable. By using multilevel fusion and transformation, the DeepSurv technique applies deep learning technology to combine various linear and nonlinear components into a linear combination in order to anticipate result events. The importance ranking measured by the DeepSurv model differs from that of the RSF model. The calculation of Permutation Importance is based on the model that has already been trained. The data of one variable in the dataset is disrupted, the other variables are kept unchanged, and the degree of change in the results is observed, giving a weighted score to that variable. DeepSurv is a deep feed-forward neural network. Compared with ordinary feed-forward neural networks, DeepSurv allow more than one hidden layer and applies modern techniques such as weight decay regularization, Rectified Linear Units (ReLU), Batch Normalization and learning rate scheduling (20). Random survival forest (RSF) is a random forest method for analyzing right-censored survival data (28). The basic structure of RSF as a decision tree-based machine learning algorithm is different from that of deep feed-forward neural networks, which should be the fundamental reason for the different results in measuring the importance of features. According to several study findings, the predictions generated using the DeepSurv model are superior to those made using conventional linear prediction models (29–31).

Our study showed advantages in discrimination and capacity compared to previous studies predicting gastric cancer survival. Wang (32) used a nomogram to fit data from gastric adenocarcinoma patients in the SEER database from 2014 to 2015 to predict O.S., with a c-index of 0.707 for the test cohort. In our study, the discrimination of the CoxPH model was slightly improved (0.755), which may be related to the fact that we included more cases. The algorithm proposed by Shapiro (33) progressed under predicting 1-year survival, with an AUC of 0.63 in the internal validation dataset (34). Although our DeepSurv model slightly outperformed the Shapiro algorithm in predicting 1-year survival (AUC of DeepSurv: 0.828), what makes our study more significant is that using the deep feed-forward neural network algorithm. Our model has an advantage over the extant prognostic models for gastric cancer patients. However, comparison with other models should be further investigated due to gaps in the selection of variables and the number of cases.

There were several restrictions placed on the current investigation. First, for the patients with gastric adenocarcinoma gathered from the SEER database, some potentially vital information was lacking, such as whether tumors were surgically removed, the kind of chemotherapy used, medications, the patient’s psychological status, religious beliefs, and level of education, as well as their family’s history of tumors. Many contemporary studies showed that perioperative chemotherapy could significantly improve progression-free and overall survival in patients with operable gastric or lower esophageal adenocarcinomas (35, 36). Using neoadjuvant chemotherapy significantly increases overall survival in complete pathologic response patients compared to neoadjuvant chemoradiotherapy (37). The performance of the current prognostic prediction model would be further improved with well-developed information. Second, the established DeepSurv prediction model was not tested using additional data; our analysis only contained data for patients with stomach adenocarcinoma who resided in certain regions of the United States. Only internal validation was performed in this study. The generalizability and accuracy of the DeepSurv model may require significant additional data for external validation. Third, while it is being built, the DeepSurv model has its intrinsic limits. Because the black-box model has hidden layers, we cannot fully comprehend the calculations made during model building or the resulting restrictions. Future research should make the necessary efforts to address the issues above.

## Conclusions

A deep learning algorithm was developed to predict more accurate prognostic information for gastric cancer patients. The DeepSurv model has advantages over the CoxPH and RSF models and performs well in discriminative performance and calibration.

## Data availability statement

The original contributions presented in the study are included in the article/**Supplementary Material**. Further inquiries can be directed to the corresponding author.

## Author contributions

YZ designed the study, and prepared the manuscript draft. FC, KL and JZ performed the data collection and analysis. JZ critically revised the manuscript for important intellectual content. All authors contributed to the article and approved the submitted version.
