# Supplementary material for: Development and validation of survival prediction model for gastric adenocarcinoma patients using deep learning: A SEER-based study
Source: Front Oncol. 2023 Mar 7;13:1131859. doi: 10.3389/fonc.2023.1131859 (PMC10029996; doi:10.3389/fonc.2023.1131859)
Supplement: Supplementary file 1 [file Image_1.pdf]

Supplementary Material

Development and validation of survival prediction model for gastric adenocarcinoma patients using deep learning: A SEER-based study

Junjie Zeng, MD[1#], Kai Li, MD[1#], Fengyu Cao, MD[1], Yongbin Zheng, MD, PhD[1]*

[1]Department of Gastrointestinal Surgery, Renmin Hospital of Wuhan University, Wuhan, Hubei, China.

[#]Junjie Zeng and Kai Li contributed equally to this work.

*Corresponding authors: Yongbin Zheng, MD, PhD, Department of Gastrointestinal Surgery, Renmin Hospital of Wuhan University, Wuhan, Hubei, 430060, China. Email: yongbinzheng@whu.edu.cn

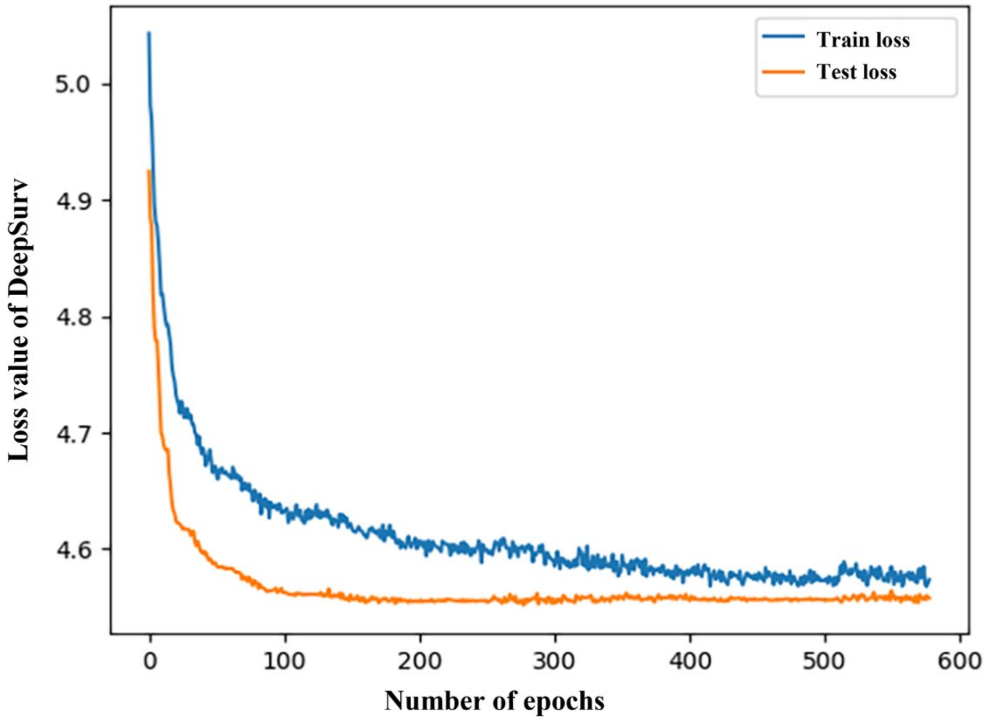

Supplementary Figure 1. The graph of the loss function for DeepSurv. The loss function after successive iterative operations is shown in the figure. The loss functions of both the training and test sets tend to be stable, and no overfitting occurs.

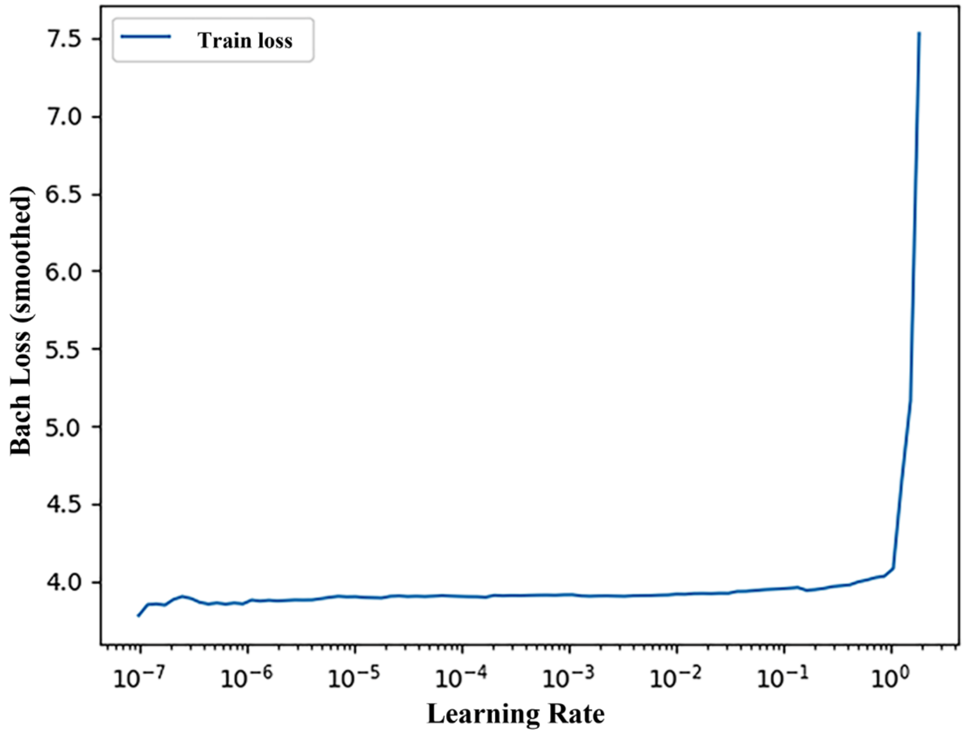

Supplementary Figure 2. The graph of the learning rate for DeepSurv. The figure shows the loss function resulting from the change in the learning rate during the iterative operation of the model. The learning rate determines how much the model parameters are adjusted in each parameter update step. As illustrated in the figure, the learning rate starts from 0.01 to more minor, and the model loss tends to stabilize.
